# The antihyperlipidemic effects of fullerenol nanoparticles via adjusting the gut microbiota in vivo

**DOI:** 10.1186/s12989-018-0241-9

**Published:** 2018-01-17

**Authors:** Juan Li, Runhong Lei, Xin Li, Fengxia Xiong, Quanyang Zhang, Yue Zhou, Shengmei Yang, Yanan Chang, Kui Chen, Weihong Gu, Chongming Wu, Gengmei Xing

**Affiliations:** 1 CAS Key Laboratory for Biomedical Effects of Nanomaterial & Nanosafety, Institute of High Energy Physics, Chinese Academy of Science (CAS), Beijing, 100049 China; 2 Pharmacology and Toxicology Research Center, Institute of Medicinal Plant Development, Chinese Academy of Medical Sciences & Peking Union Medical College, Beijing, 100193 China

**Keywords:** Fullerenols, Gut microbiota, Short-chain fatty acid (SCFA), Blood lipids, Butyrate-producing bacteria

## Abstract

**Background:**

Nanoparticles (NPs) administered orally will meet the gut microbiota, but their impacts on microbiota homeostasis and the consequent physiological relevance remain largely unknown. Here, we describe the modulatory effects and the consequent pharmacological outputs of two orally administered fullerenols NPs (Fol1 C_60_(OH)_7_(O)_8_ and Fol113 C_60_(OH)_11_(O)_6_) on gut microbiota.

**Results:**

Administration of Fol1 and Fol113 NPs for 4 weeks largely shifted the overall structure of gut microbiota in mice. The bacteria belonging to putative short-chain fatty acids (SCFAs)-producing genera were markedly increased by both NPs, especially Fol1. Dynamic analysis showed that major SCFAs-producers and key butyrate-producing gene were significantly enriched after treatment for 7–28 days. The fecal contents of SCFAs were consequently increased, which was accompanied by significant decreases of triglycerides and total cholesterol levels in the blood and liver, with Fol1 superior to Fol113. Under cultivation in vitro, fullerenols NPs can be degraded by gut flora and exhibited a similar capacity of inulin to promote SCFA-producing genera. The differential effects of Fol1 and Fol113 NPs on the microbiome may be attributable to their subtly varied surface structures.

**Conclusions:**

The two fullerenol NPs remarkably modulate the gut microbiota and selectively enrich SCFA-producing bacteria, which may be an important reason for their anti-hyperlipidemic effect in mice.

**Electronic supplementary material:**

The online version of this article (10.1186/s12989-018-0241-9) contains supplementary material, which is available to authorized users.

## Background

Nowadays, an increasing body of nanomaterials are developed as new therapeutic and diagnostic tools for many serious diseases [1]. The prominent therapeutic effects of nanodrugs on cancers [2], angiogenesis inhibition [3] antiinflammation [4] and other conditions have been demonstrated through animal, cellular, and biomolecular experiments [5, 6]. Oral administration is a preferred route for clinical compliance of nanodrugs which increases the likelihood that nanomaterials encounter microbes in the gastrointestinal (GI) tract. Therefore, what occurs when nanomaterials meet gut microbes is an important issue worthy of exploring.

There are more than 100 trillion microbes living in our digestive tracts which are collectively called the gut microbiota. A growing body of evidence indicates that the gut microbiota is indispensable to the maintenance of human health [7]. Structural changes in the gut microbiota are closely associated with various diseases, especially metabolic disorders such as obesity, insulin-resistant diabetes, and nonalcoholic fatty liver disease [8, 9]. Recent investigations revealed that the therapeutic effects of many medicines such as metformin [10], berberine [11], and *Ganoderma lucidum* [12] are at least partially attributable to their modulation of gut microbiota, demonstrating the powerful capacity of the gut microbiome to mediate the safety and efficacy of traditional oral drugs [13, 14]. The impacts of nanoparticles (NPs) on the gut microbiota have also attracted increasing attention. The nanoparticles of titanium dixoide (TiO_2_), Zinc and silver have been reported to be able to change the gut microbial community, but their modulating effects on the gut microbiota remain controversial among different research groups [15–20].

Fullerenols are polyhydroxylated C60 with excellent properties such as easy to be obtained, high water solubility and good biocompatibility [4, 21, 22]. Fullerenol NPs are now widely used in biological and medical researches due to their various biological activities. Previous studies have shown that fullerenol NPs can inhibit the growth of various microbes in vitro [22, 23], but their in vivo impact on gut microbes is largely unknown. Therefore, fullerenol NPs are a suitable condidate to investigate the modulating role of nanomaterials on the gut microbiota and the consequent physiological relevance.

In the present study, we investigated the impacts of two fullerenol NPs (Fol1 C_60_(OH)_7_(O)_8_ and Fol113 C_60_(OH)_11_(O)_6_) on gut microbiota homeostasis in vivo. We also assessed the influences of fullerenol NPs on the SCFA-producing bacteria levels, fecal SFCA concentration, and lipid levels in blood and liver to establish a physiological association between the gut microbe-regulating roles of fullerenols and their pharmacological benefits. Lastly, we examined the impacts of fullerenol NPs on SCFA-producers in vitro and compared them with those of inulin to clarify how fullerenols NPs modulate the gut microbiome and exert antihyperlipidemic effects.

## Methods

### Preparation of fullerenols

Fullerenols (Fol 1) were synthesized by the alkaline reaction [24]. Briefly, a solution of C_60_ in toluene was added to an aqueous solution containing NaOH and 40% tetrabutylammonium hydroxide (TBAH) as catalyst. The mixture was stirred at room temperature for 24 h. The color of the solution changed from deep violet to colorless, while a brown sludge precipitated on the bottom of the beaker. The aqueous phase was then separated and evaporated under vacuum to obtain the crude product, which was further washed by methanol. The crude product was then passed through a Sephadex G-25 column and eluted with double distilled water. The difference of the preparations of Fol 113 was used H_2_O_2_ 30% was as initiators in the stirring mixture.

Purified fullerenols were prepared by a previously reported method in our laboratory [24]. Dry samples were analyzed on a Nicolet Magna-IR750 FTIR spectrophotometer equipped with a Nic-plan IR microscope. Scanning electron microscope (SEM) imaging was performed using a HITACH 4800 s (Japan) instrument. Dynamic light scattering(DLS) was monitored the hydrodynamic sizes and zeta potentials of fullerenol nanoparticles in saline, simulated gastric and intestinal solusion by Malvern Zetasizer Nano ZS instrument at the concertration of 500 μg/mL and the solusions were prepared according to National Formulary [25]. The samples were deposited onto silica substrates for SEM and onto high-purity gold substrates for XPS. Electronic properties of the samples were studied by XPS at the photoelectron station of Beijing Synchrotron Radiation Facility of the Chinese Academy of Sciences. The XPS measurements in an ultra-vacuum chamber with background pressure ranging 8 × 10^−10^ to 1 × 10^−9^ Torr and photon energy of 400.0 eV. The assay resolution was estimated to be ~0.5 eV. All electron spin resonance (ESR) experiments were carried out by using a JES-FA200 X-band ESR spectrometer (JEOL, Japan), at Tsinghua University in China.

### Modulation of gut microbiota by fullerenols in mice

All the animal experiments were performed in accordance with the National Institutes of Health regulations for the care and use of animals in research and were approved by the Medical Ethics Committee of Peking Union Medical College (No. YZS201603004).Twenty-four male specific pathogen-free (SPF) C57BL/6 mice (8-week-old, 22–26 g) were obtained from Vital River Laboratory Animal Technology Co., Ltd. (Beijing, China). Animals were kept in a humidity-controlled room on a 12-h light-dark cycle with food and water available ad libitum for 1 week. The mice were then divided randomly into 3 groups (8 animals per group) and fed with normal diet. The Fol1 and Fol113 groups were gavaged with Fol1 or Fol113 NPs (20 mg/kg per day, suspended in distilled water) for a month, while the control group (NC) was given an equal volume of distilled water. Fresh feces were collected from each mouse at 2 h after the last gavage, snap-frozen in liquid nitrogen then stored at −80 °C for subsequential analysis.

### Fullerenol NPs distribution and excretion in mice

The In vivo distribution of fullerenol NPs were measured by PET imaging using ^64^Cu-labeled fullerenol NPs (^64^Cu-C_60_) as described in previous study [26]. The ^64^Cu-C_60_ nanoparticles (25.9 MBq) dispersed in 100 μL saline were given to the mice by single oral administration (*n* = 3). The mice were anesthetized with 1.5% isoflurane for imaging at 0.5, 1, 2 and 6 h. The data were acquired using a small animal PET scanner (Eplus-166, Institute of High Energy Physics, Chinese Academy of Sciences). The PET images were corrected for detector efficiency, dead-time, decay, photon scatter, and attenuation. The control groups were injected with 25.9 MBq ^64^CuCl_2_ and subjected to the same PET protocol.

To assess the excretion of fullerenol NPs, another animal experiment was performed as described above. Animals were given ^64^Cu-C_60_ nanoparticles (25.9 MBq) dispersed in 100 μL saline by a single oral administration (*n* = 3). These animals were kept in metabolism cages for 24 h after material gavage. Feces and urine samples were collected at 2, 4, 6 and 24 h and counted in a CAPRAC-t well-type counter (Capintec, Inc., Pittsburgh, PA, USA).

### In vitro single-batch fermentation

Fresh fecal materials were obtained from healthy C57 mice, pooled together, and suspended in 0.1 M sterile PBS (pH 7.0) to prepare a 10% *w*/*v* fecal slurry. An aliquots of 5 mL fecal slurry was inoculated into each 100 mL fermentation vessel that contained 15 mL of the modified Broth medium (Hopebio Ltd., China). The Fol 1 and Fol 113 NPs were added into the vessels with a final concentration of 0.1 mg/mL. As positive control, inulin, a well-known prebiotics that is able to promote short-chain fatty acid (SCFA)-producing bacteria [27], was added into the vessels to achieve a final concentration of 5 g/L. The cultures were fermented for 48 h in the micro-anaerobic incubation system (INVIVO 400, Ruskin Technologies, UK). After culture, the bacteria were collected by 12,000 rpm centrifugation for 20 min. The cell pellets were used for MALDI-TOF-MS and real-time quantitative PCR analysis. The experiment were performed in triplet.

### Phylogenetic analysis

Fecal DNA samples of each mouse extracted using a FastDNA® Spin Kit for Stool (MP Biomedicals, Santa Ana, USA) was then amplified by barcoded composition primers flanking the V3/V4 regions of the 16S rRNA gene. The composite primers consist of BCP-F (50-GCCTTGCCAGCCCGCTCACACTCCTACGGRAGGCAGCAG-30; the underlined sequence indicates the target region) and BCP-R (50-GCCTCCCTCGCGCCATCAG-X-TACNVGGGTATCTAATCC-30; “X” denotes the unique barcode for each sample). The PCR reaction was performed as follows: 95 °C for 5 min, followed by 30 cycles of 95 °C for 30 s, 55 °C for 30 s and 72 °C for 30s, with a final extension at 72 °C for 5 min. The amplicons were pooled and purified using a QiaQuick PCR purification kit (Qiagen, Valencia, USA). The mixed PCR products were sequenced on a 454 FLX pyrosequencer platform (Roche, Branford, CT) according to manufacturer’s instruction. Pyrosequencing data analysis was carried as previously described [28].

### SCFA analysis

Fecal SCFAs were evaluated by a gas chromatography mass spectrometer (GC-MS). Briefly, 200 mg of frozen feces was suspended in 1 mL of 1% HCl and strongly vortexed for 1 min. 2-Ethylbutyric acid (Sigma Aldrich, USA) was added as internal standard in a final concentration of 2 mM. The samples were centrifuged at 5000×g for 5 min and the supernatant was acidified to pH 0 with HCl (10 mol/L). Each sample was extracted at 4 °C using an equal volume of diethyl ether. Aliquots (80 μL) of extracts were added with 16 μL N-tert-butyldimethylsilyl-N methyltrifluoroacetamide (Sigma Aldrich) and incubated at 40 °C for 2 h. The SCFAs contents of each samples were analyzed on an Agilent 5975C GC-MS (Agilent Technologies, Palo Alto, CA, USA) equipped with a HP-5MS column (0.25 mm × 30 m × 0.25 μm) and a 5973 Network Mass Selective Detector. The GC program was performed as follows: started at 40 °C, heated to 70 °C by 5 °C /min and held for 3.5 min, then ramped at 20 °C/min to 160 °C followed by 35 °C /min to 280 °C and held for 3 min. The m/z ratios of monitored ions were as follows: 117 (acetate), 131(propionate), 145 (butyrate), and 173 (2-ethylbutyric acid). SCFAs were quantified with a five-point calibration curve.

### Dynamic modulation of microbiota and lipids in body by fullerenols

C57BL/6 male mice (8-week-old) were kept in a humidity-controlled room on a 12-h light-dark cycle with food and water available ad libitum. The mice were then divided randomly into 3 groups (8 animals per group) and fed with high-fat diet (HFD) which contained 60% fat, 14% protein, and 26% carbohydrate and provided a total energy content of 21.0 kJ/g. Fol1 and Fol113 NPs (20 mg/kg per day, suspended in distilled water) was administrated via oral gavage for 3 weeks. The control group was given equal volumn of distilled water only. Fecal samples were taken from each mouse on 0, 3, 7, 14 and 28 day after fullerenols treatment. Total bacteria number and the relative abundance of specific bacteria (*Allobaculum spp.*, *Clostridium cluster IV* and *XIVa*) as well as the relative abundance of butyrate-producing genes (BcoA and Buk) in the fecal were determined as described below. After the last fecal samples were acquired, mice were anesthetized in chambers saturated with isoflurane and sacrificed by cardiac puncture. Blood and liver tissue were taken for the measure of serum and liver levels of triglycerides (TG) and total cholesterol (TC) by respective kits (BioSino Co., Ltd., Beijing, China).

### Realtime PCR

Real-time quantitative polymerase chain reaction (realtime PCR) was performed on a 7500 Real-Time PCR System (Applied Biosystem) to quantify the abundance of specific bacteria and butyrate-producing genes. The gene-specific primers were designed to target the total, specific bacteria (Allobaculum spp., Bifidobacterium spp., Clostridium clusters IV and XIVa), butyrate-producing genes (butyryl coenzyme A transferase (BcoA) and butyrate kinase (Buk)) (Additional file 1: Table S2). Cloned 16S rRNA genes of *E. coli* were used to construct standard curve for total bacteria copies. The total bacteria were expressed as log10 copies (16 S DNA gene)/g feces. The relative abundance of specific bacteria or functional genes was normalized to the total bacteria.

### Histologic analysis

A piece of liver was taken from each animal and fixed in 4% buffered neutral formalin for at least 2 days. Then liver samples were embedded in paraffin and cut at 4 μm. The sections were stained with hematoxylin and eosin (H&E), and their morphological changes were evaluated. The steatotic level of liver was assessed by the fatty degeneration (ballooning) of hepatocytes. At least 15 slides of each group were analyzed.

### Quantitative analysis of fullerenols by MALDI-TOF-MS

The polyhydroxyl C_70_ were as reference to quantify the fullerenols of C_60_ by MALDI-TOF-MS without matrix (Matrix-Assisted Laser Desorption/Ionization Time of Flight Mass Spectrometry) (UltrafleXtreme, Bruker, Germany). The standard curve of C_60_/C_70_ and the concentration of C_60_ were obtained and the bacteria suspension samples were analysed under the same condition.

### Statistics

Data are presented as the means ± SEM. SPSS 17.0 software was used for the statistical analysis. To analyze the correlation between the abundance of different gut microbe and SCFAs level, multiple-testing corrected pairwise Spearman correlation analysis was performed on the relative abundance of individual genus and the respective fecal SCFA content in each animal, as described previously [29]. The significance of group differences for normally distributed data was assessed by one-way ANOVA followed by Tukey post hoc tests. *P* < 0.05 was considered statistically significant.

## Results and discussion

### Physicochemical characterization of fullerenol NPs

**Fig. 1:**
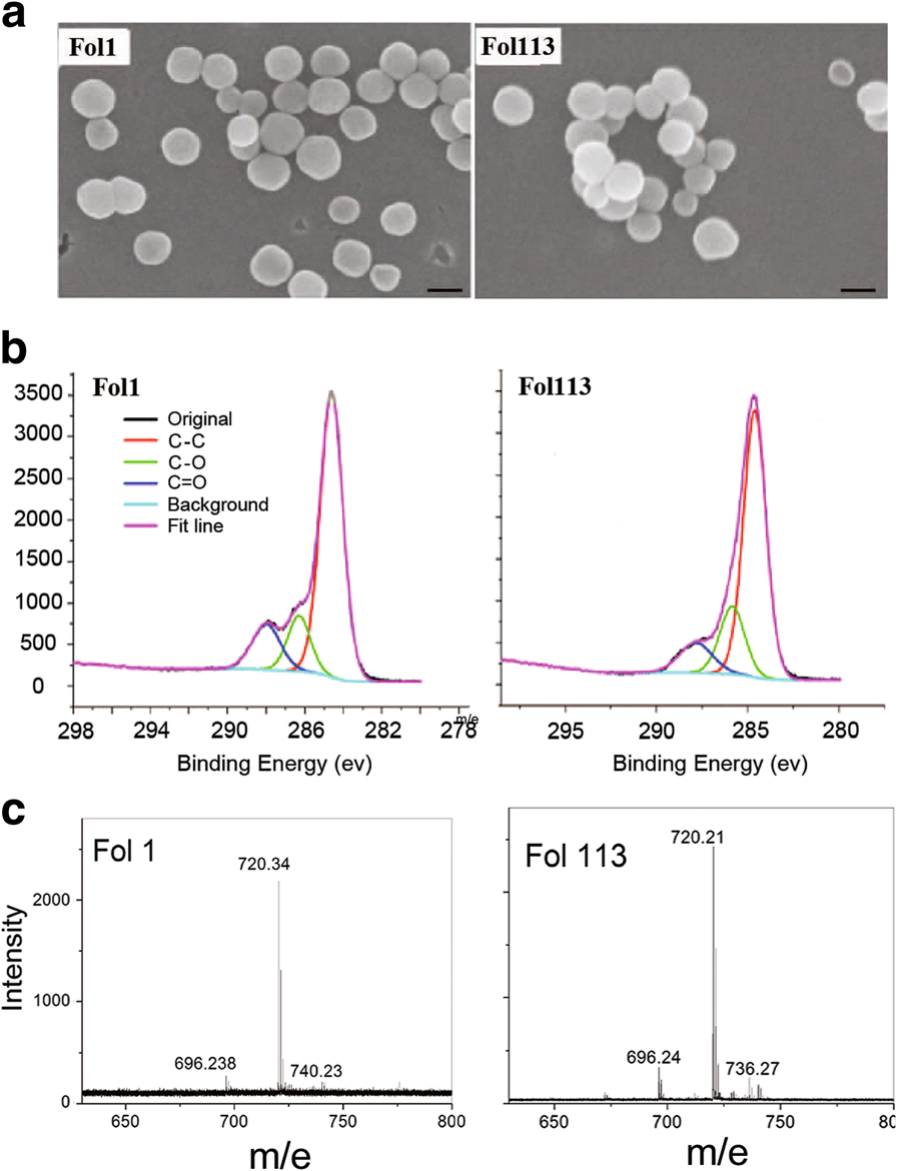
The physiochemical characterizations of fullerenols. **a** SEM images of NP aggregates (bar = 100 nm), **b** XPS data, **c** MALDI-TOF-MS data without a matrix

Fol1 and Fol113 NPs were synthesized by different routes and purified using an adapted method previously established in our laboratory [24]. Both NPs are stable in aqueous solution for several months (Additional file 1: Figure S1a). Scanning electron microscopy (SEM) images showed that Fol1 and Fol113 have aggregation states, with d = 100 ± 13 nm and 90 ± 5 nm, respectively (Fig. 1a). The hydrodynamic sizes of Fol113 NPs were also smaller than Fol1 as determined by dynamic light scattering experiments (Additional file1: Table S1) in saline, simulated gastric and intestinal solutions. The zeta potentials and hydrodynamic sizes of Fol1 and Fol113 indicated good stability in the GI tract. The ultraviolet and Fourier-transform infrared spectroscopy (FTIR) data in Additional file1: Figure S1 indicated that the fullerene cages have similar groups and structures. However, the X-ray photoelectron spectroscopy (XPS) data (Fig. 1b) indicated different types of functionalized carbon atoms on the carbon cages. With a general formula of C_60_(OH)m(O)n, Fol1 should be C_60_(OH)_7_(O)_8_ and Fol113 should be C_60_(OH)_11_(O)_6_ according to the XPS data (Table 1). The characteristic fullerene peak of 720 (m/e) was detected in both fullerenol NPs on MALDI-TOF-MS which kept the features of fullerenes (Fig. 1c).

**Table 1:** Comparison of relative peak areas in the XPS spectra of fullerenols

NPs	C-C (%)	C-O (%)	C=O (%)	Formula (C60(OH)m(O)n) (calculated)
Fol 1	71.66	13.97	14.37	C60(OH)7(O)8
Fol 113	69.31	19.30	11.39	C60(OH)11(O)6

### Fullerenol NPs markedly change the gut microbiota structure

To assess the impacts of fullerenol NPs on gut microbiota, C57BL/6 mice were treated with Fol1 or Fol113 NPs (20 mg/kg/day, oral administration) for 28 days, and distilled water-treated mice were used as a negative control (NC). Fresh feces samples were collected from each mouse within 2 h after the last gavage and sent for 16S rRNA pyrosequencing. The bar-coded pyrosequencing provided 1,150,372 usable pyrosequencing reads (189,302 operational taxonomic units [OTUs]) from 24 fecal samples. After discarding the singleton OTUs that could have been due to sequencing error, 20,750 non-singleton OTUs (3537 ± 855 OTUs per sample) were recovered for phylogenetic analysis. Rarefaction and Shannon-Wiener curves showed that although new, rare phylotypes would be expected with additional sequencing, most gut microbial diversity was captured (Additional file 1: Figure S2a and S2b).

**Fig. 2:**
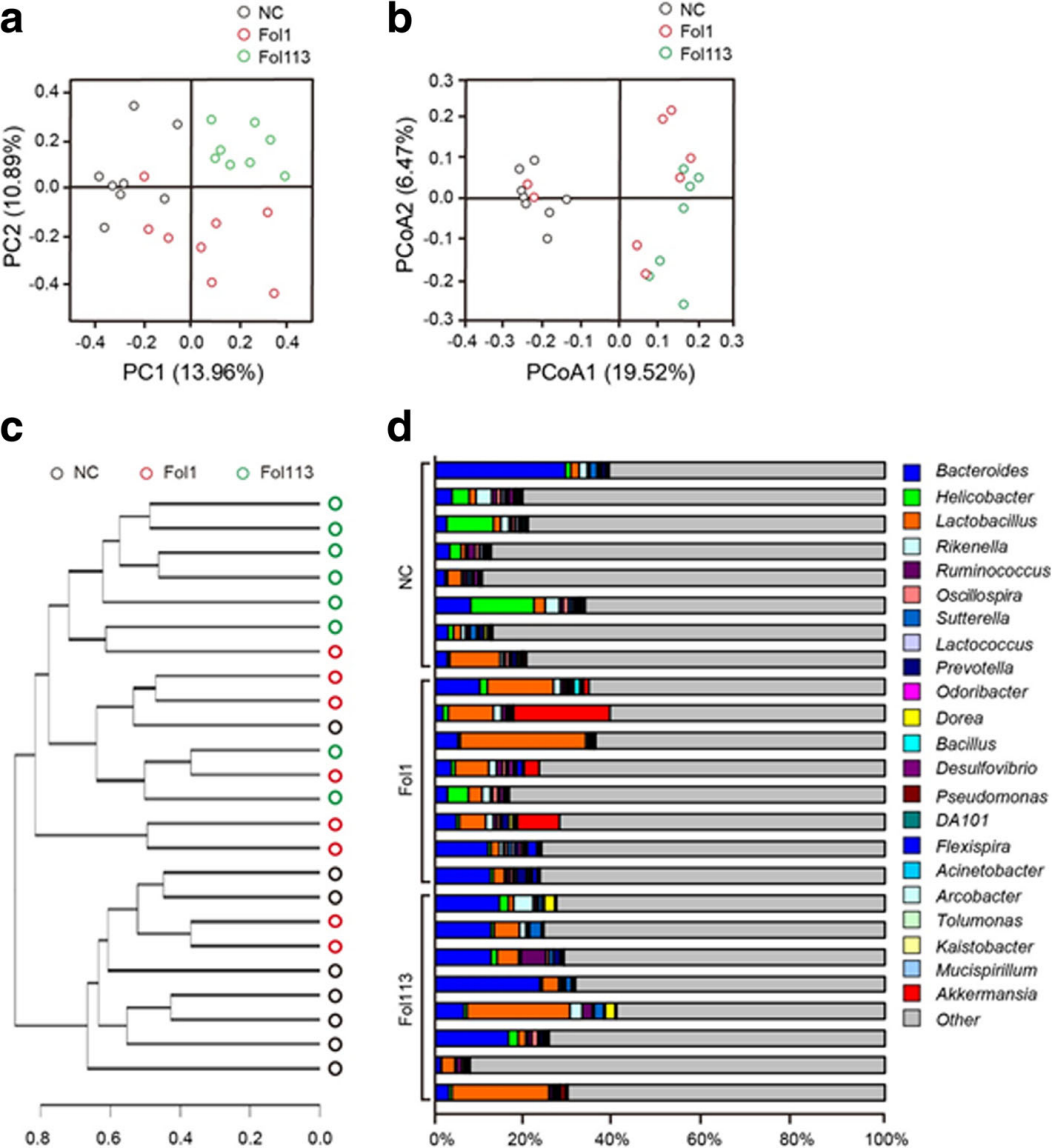
Responses of the gut microbiota structures to fullerenol NPs. **a** PCA score plot, **b** PCoA score plot, **c** clustering of the group means based on the Mahalanobis distances calculated using MANOVA about unweighted UniFrac PcoA, **d** proportion of the 22 most abundant genera in each animal

Oral administration of Fol1 or Fol113 NPs for 28 days remarkably shifted the overall structure of gut microbacteria in vivo. Firstly, principal component analysis (PCA) (Fig. 2a), principal coordinate analysis (PcoA) (Fig. 2b), and UniFrac distance-based hierarchical clustering analysis (Fig. 2c) were used to monitor overall structural changes in the gut microbiota. The results show that both fullerenol NPs markedly shifted structure. Most samples of Fol1 and Fol113 groups were completely separate from the control group along Principal component/coordinate 1 (PC1/PCoA1) (*P* < 0.05) except for a few overlaps (Fig. 2a and b). Hierarchical clustering analysis also revealed remarkable modulatory effects of the fullerenol NPs on gut microbiota structure. The gut flora modulated by Fol1 and Fol113 were largely separate from the NC group, but there were some overlaps (Fig. 2c).

Venn diagram analysis confirmed marked separation of the gut microbes by Fol1 and Fol113 NPs at the OTU level. As shown in Additional file 1: Figure S3, there were a total of 4328 OTUs in all three groups. Those treated with Fol1 or Fol113 NPs shared 8707 (4379 + 4328) and 6547 (2219 + 4328) OTUs with the NC group, respectively. However, there were 6423 OTUs exclusive to NP-treated animals (Additional file 1: Figure S3). Additional file 1: Figure S4 displays the relative abundance of the top 50 OTUs in each animal, and NPs enriched those belonging to the genera *Lactobacillus*, *Akkmansia*, and *Allobaculum*. As the three genera are known to be beneficial for lipid metabolism, this suggested that Fol1 and Fol113 NPs could be useful for treating metabolic diseases such as obesity and hyperlipidemia.

Taxon-based analysis also revealed large changes in gut microbial composition induced by Fol1 and Fol113 NPs. A total of 17 in 42 phyla, 52 in 122 classes, 86 in 236 orders, 173 in 420 families, and 252 in 727 genera were significantly different among the NC and NP-treated groups (*P* < 0.05, Additional file 2: Table S3). Although both fullerenol NPs remarkably changed the relative abundance of numerous types of bacteria, the significantly changed phylotypes accounted for only a minor part of the total gut microbiota. The five phyla (Acidobacteria, Gemmatimonadetes, Nitrospirae, TM6, and Thermi) significantly changed by both fullerenols NPs represented 0.602% of the total gut bacteria in the NC group. For major phyla in the gut microbiome, Fol1 and Fol113 NPs exerted minimal influence on the abundance of Firmicutes and Bacteroidetes, but both moderately decreased the relative abundance of Proteobacteria that is known to contain multiple opportunistic pathogens (Additional file 2: Table S3). At the genus level, the two fullerenols showed enriching effects on *Lactobacillus*, *Dorea*, *Bifidobacterium*, *Allobaculum*, *Blautia*, *Parabacteroides*, *Akkermansia*, and *Anaerotruncus* (Fig. 2d and Additional file 2: Table S3), all of which are known SCFA producers.^26^ Interestingly, although none of the eight SCFA-producing genera was significantly enriched by either fullerenol NP, their total abundance in each animal was significantly increased by Fol1 NPs (17.30% vs 3.45%, *P* < 0.05) and almost significantly by Fol113 NPs (10.63% vs 3.45%, *P* = 0.077). Although further investigations, especially the dose-dependent effects of fullerenol NPs on the gut microbiota, are still needed, these results provided clear evidence that fullerenol NPs can modulate gut microbiota.

**Fig. 3:**
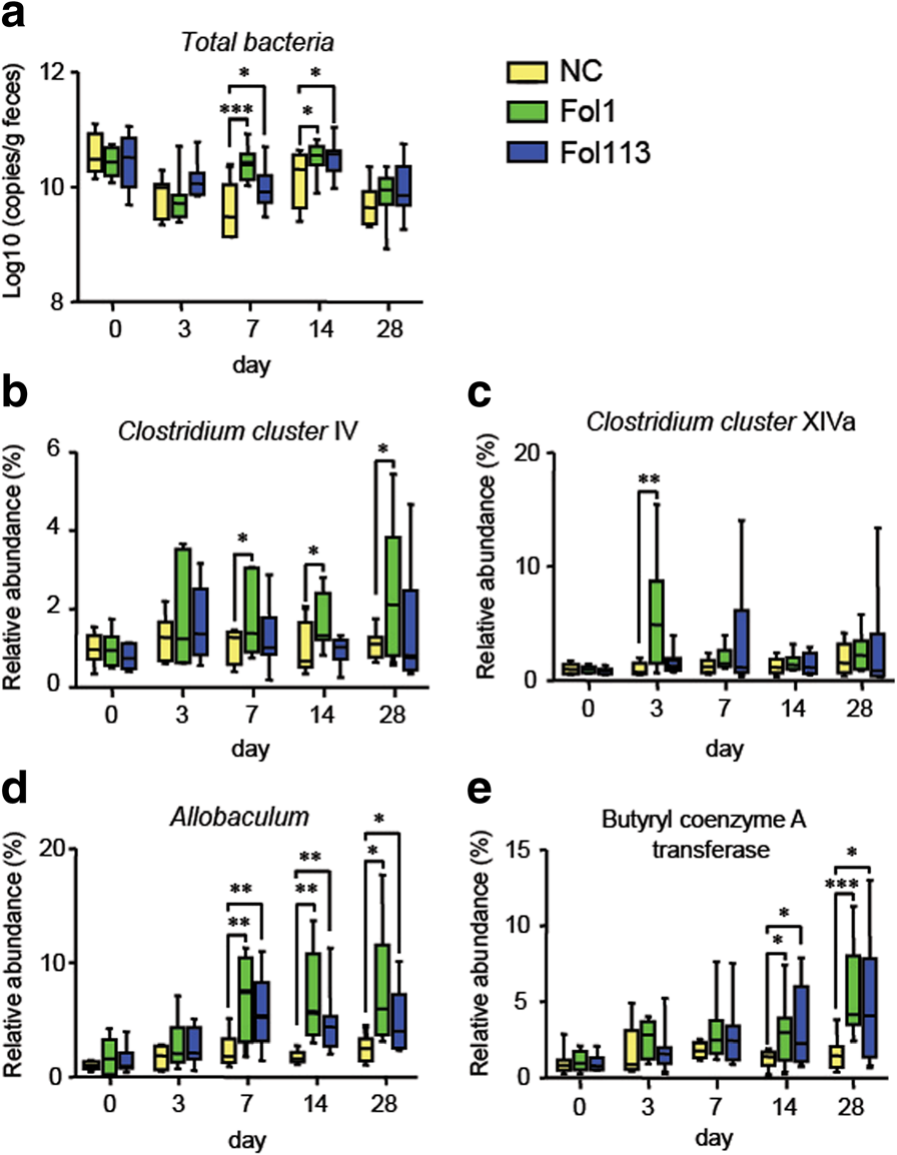
Dynamic analysis of total bacteria copies (**a**), relative abundance of *Clostridium* IV (**b**), *Clostridium* XIVa (**c**), *Allobaculum* spp. (**d**) and butyryl CoA transferase (BcoA) (**e**) in feces. ^*^P < 0.05, ^**^P < 0.01, ^***^P < 0.001 vs NC

Dynamic analysis showed that in addition to changing the static state of gut microbiota, Fol1 and Fol113 NPs modulated microbial composition dynamically. The dynamic experimental groups were treated with Fol1, Fol113, or distilled water, and fecal samples from each mouse were collected on day 0, 3, 7, 14, and 28. Total bacteria number, the relative abundance of SCFA-producing bacteria (*Allobaculum* spp., *Clostridium cluster IV*, and *XIVa*), and the relative abundance of butyrate-producing gene (butyryl coenzyme A transferase [*BcoA*]) were determined by real-time quantitative polymerase chain reaction. Compared to NC, oral administration of Fol1 and Fol113 NPs did not remarkably decrease the total numbers of gut bacteria but increased SCFA-producing bacteria such as *Clostridium* clusters *IV* and *Allobaculum* spp., as well as the relative abundance of BcoA after treatment for 7 days (Fig. 3a-e). These results were in accordance with 16S rRNA sequencing and indicate that the modulatory effects of fullerenol NPs on SCFA producers and genes are time dependent.

### Fullerenol NPs enhance fecal SCFA content and decrease blood and liver lipids

**Fig. 4:**
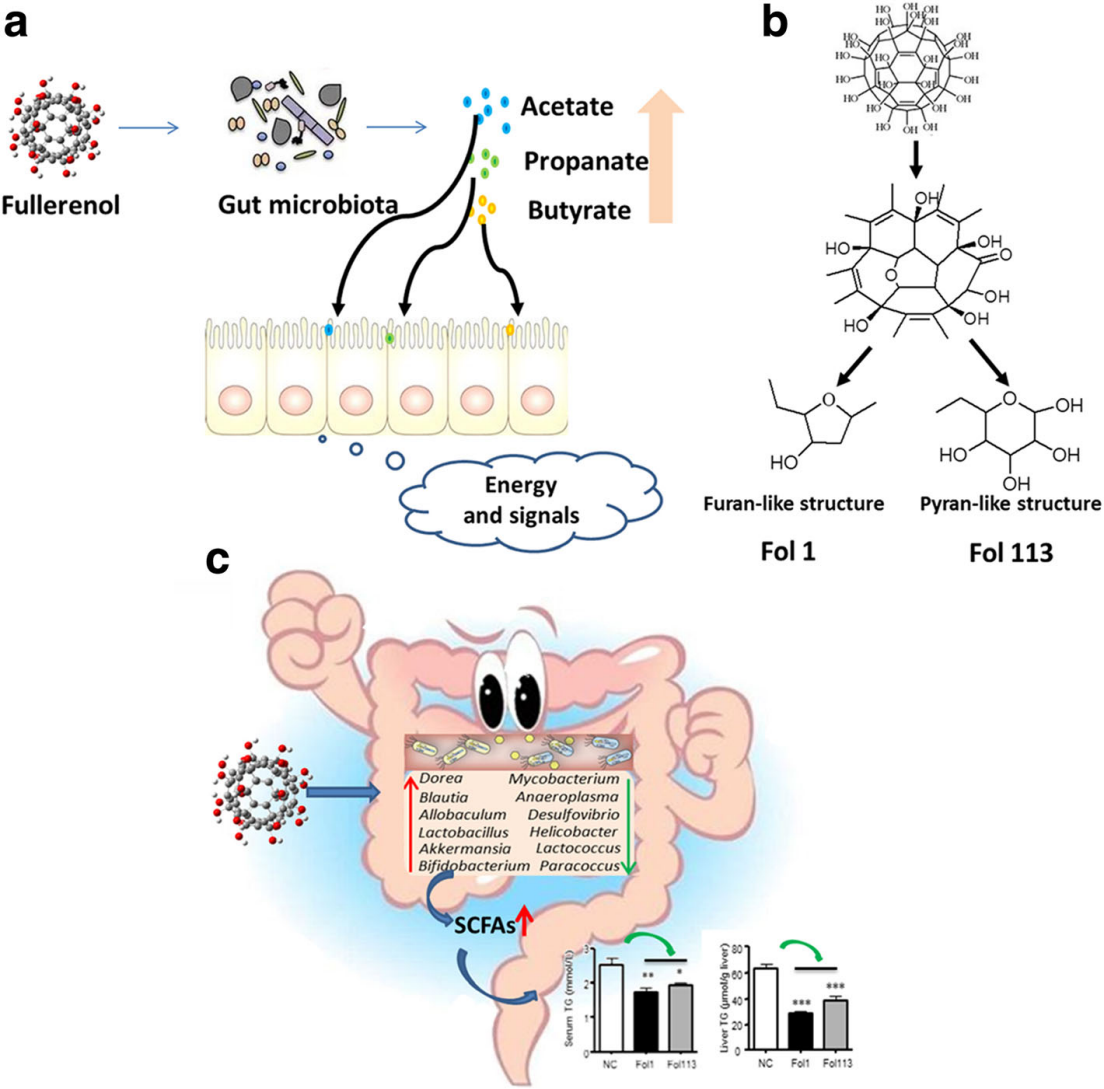
**a** Fullerenol NPs ameliorate hyperlipidemia by modulating gut microbiota structure and increasing SCFA production. **b** Fullerenol NPs contain furan- and pyran-like structures that could be used by gut microbes

**Fig. 5:**
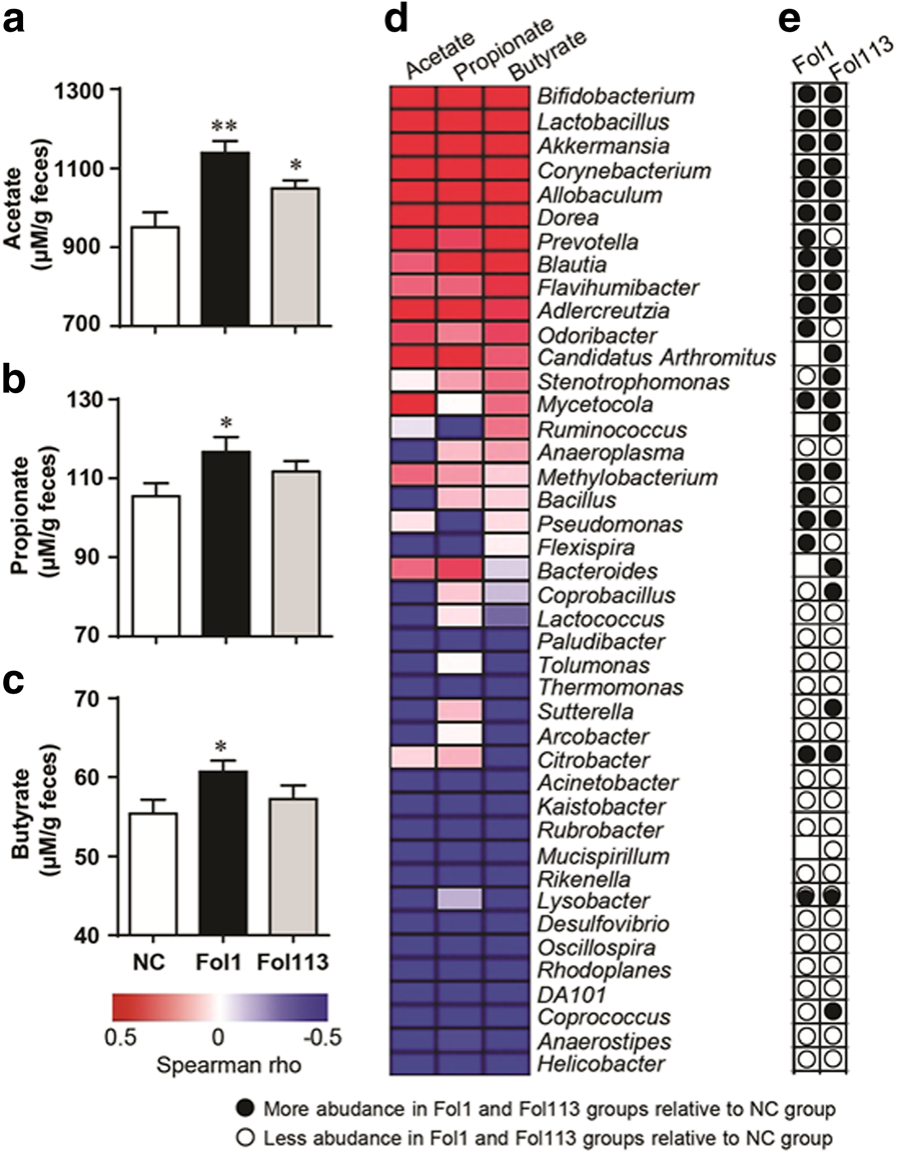
Fullerenol NPs alter SCFA fecal concentrations in mice. Levels of acetate (**a**), propionate (**b**), and butyrate (**c**) are shown. (**d**) Heatmap of correlations between metagenomics genera for fecal SCFA levels. (**e**) Influence of fullerenol NPs on the relative abundance of SCFA-related genera. White and black circles indicate decreased and increased OTUs, respectively, in the Fol1- and Fol113-treated groups compared to control. Blank indicates similar abundance between fullerenol-treated and control groups. ^*^P < 0.05, ^**^P < 0.01 vs NC

SCFAs are predominantly byproducts of the fermentation of non-digestible dietary fibers through the action of intestinal anaerobic bacteria (see Fig. 4a). Acetate, propionate, and butyrate represent the most abundant (≥95%) metabolite molecular species of intestinal SCFAs [30]. As the total abundance of main SCFA-producing genera was markedly enriched by both NPs, especially Fol1, we determined fecal acetate, propionate, and butyrate levels by gas chromatography-mass spectrometry. The fecal contents of all three were significantly increased by Fol1 NPs as compared with the NC group (Fig. 5a-c). Fol113 NPs exerted a similar enriching effect on the three major SCFAs, but the increases were not statistically significant except for acetate (Fig. 5a-c).

We further performed correlation analysis on the relative abundance of individual genera in each animal with their respective fecal SCFAs contents. The abundance of genera *Lactobacillus*, *Allobaculum*, *Bifidobacterium*, *Dorea*, and *Blautia* strongly positively correlated with fecal SCFAs levels (Fig. 5d), which was in accordance with their known SCFA-producing functions. Treatment with fullerenol NPs enriched all five genera (Fig. 5e), suggesting that the increases of *Lactobacillus*, *Allobaculum*, *Bifidobacterium*, *Dorea,* and *Blautia* might be linked to the SCFA-promoting effect of fullerenol NPs. In addition to promoting SCFA-producing microbe proliferation, the fecal SCFA concentration was also significantly increased by Fol1, and to a lesser extent by Fol113.

**Fig. 6.**
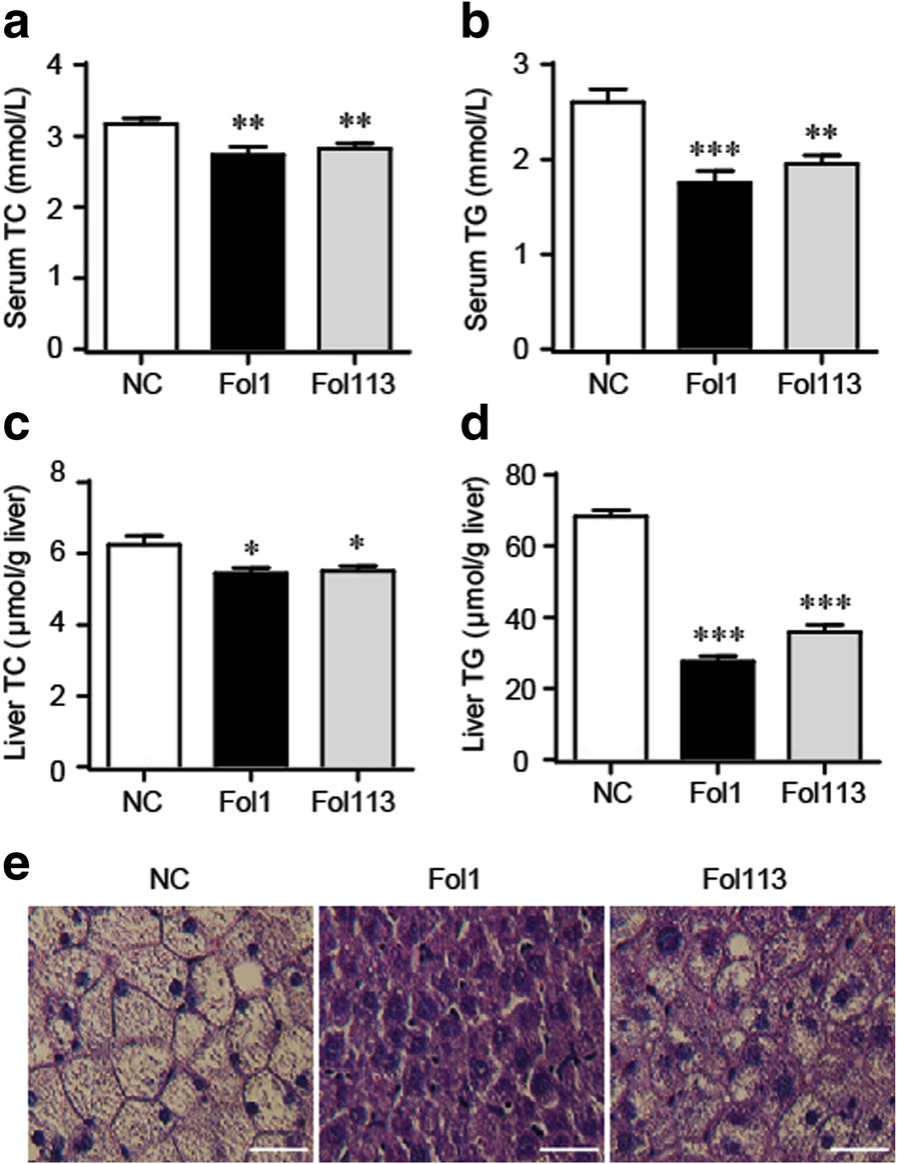
Fullerenol NPs decrease blood and liver lipids in mice. Blood levels of TC (**a**) and TG (**b**), liver levels of TC (**c**) and TG (**d**), and representative hematoxylin and eosin staining of the liver (**e**) are shown. Bar = 50 μm. ^*^P < 0.05, ^**^P < 0.01, ^***^P < 0.001 vs NC

SCFAs, especially butyrate, are key regulators of host energy homeostasis and help prevent adiposity and hyperlipidemia. To confirm the correlation between gut microbiota modulation by fullerenol NPs and pharmacological outcomes, we evaluated the effects of Fol1/Fol113 NPs on blood and liver lipid levels. In accordance with their SCFA-promoting function, treatment with Fol1 or Fol113 NPs significantly decreased serum and liver levels of total cholesterol (TC) and triglycerides (TG), with Fol1 being more effective (Fig. 6a–d). Accordingly, the high-fat diet (HFD)-induced fatty degeneration (ballooning) of hepatocytes was largely attenuated by both NPs (Fig. 6e), suggesting that Fol1/Fol113-elicited SCFA production can help prevent HFD-induced hyperlipidemia and liver steatosis.

### Fullerenol NPs show a similar capacity of inulin to promote SCFA-producing bacteria in vitro

To explore how Fol1 and Fol113 NPs promote SCFAs-producing bacteria survival, we performed a set of investigations in which we analyzed fullerenol NP structure, determined their degradation by gut flora, and carried out in vitro fermentation to assess their direct impacts on gut microbiota.

As revealed by synchrotron radiation XPS analysis and FTIR data (Fig. 1 and Additional file 1: Figure S1), fullerenol NPs were rich in complex -OH groups including peroxo groups to hydroxy groups and peroxo groups to epoxy groups [31, 32]. Fol1 and Fol113 can be expressed by the general formulae C_60_(OH)_7_(O_)8_ and C_60_(OH)_11_(O)_6_, respectively. There may be furan- and pyran-like structures on fullerenol stereostructures (see Fig. 4b). Since the furan- and pyran-like structures of fullerenol NPs appeared similar to those of the polysaccharides in dietary fiber, which is the main nutrient for SCFAs-producing gut bacteria [33], we hypothesize that Fol1 and Fol113 NPs might function like dietary polysaccharides to promote SCFAs-producing bacteria survival.

**Fig. 7:**
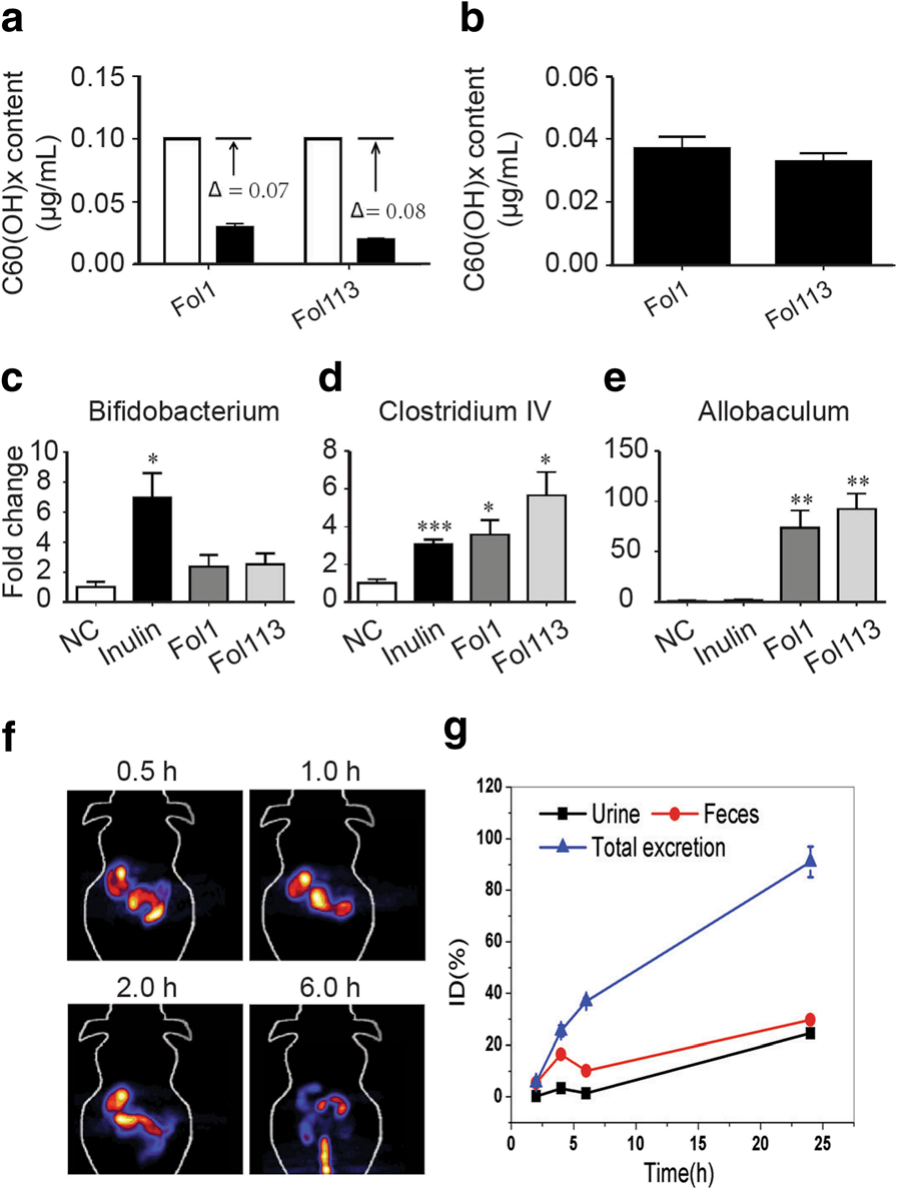
Fullerenol NPs interact with gut microbiota during in vitro gut flora fermentation. **a** Fullerenols concentration changes in the fermentation solution; **b** effects of the final concentrations of different fullerenols after in vitro fermentation; **c**-**e** responses of *Bifidobacterium, Clostridium IV*, and *Allbaculum* spp. to inulin and fullerenol NPs; ^*^P < 0.05, ^**^P < 0.01, ^***^P < 0.001 vs NC. **f** PET images of ^64^Cu- C_60_ at 0.5, 1, 2, and 6 h after single oral administration. **g** Excretion curves of ^64^Cu-C_60_ at 1, 2, 4, 6, and 24 h after single oral administration. All data points represent three animals per group. The total excretion was the sum of percentages of ^64^Cu in all the waste (∑_0-t_urine + ∑_0-t_ feces) during 0-t not at the time point

Being bacterial nutrients, NPs should be degraded by gut microbes. We therefore incubated the two fullerenol NPs with gut flora and determined whether they could be degraded by gut microbial community. We performed matrix-assisted laser desorption/ionization-time of flight-mass spectrometry (MALDI-TOF-MS) to quantify the fullerenols of C_60_ (e/m = 720) with polyhydroxyl C_70_ (e/m = 840) as reference (Additional file 1: Figure S5). After fermentation for 48 h, the concentrations of the two fullerenols were reduced by 0.069 ± 0.0031 and 0.088 ± 0.0012 mg/mL, respectively (Fig. 7a). The final concentrations of Fol1 and Fol113 in fermented solution corresponded to 0.037 ± 0.0031 and 0.033 ± 0.0017 mg/mL (Fig. 7b). Incubating fullerenols with the medium without feces for 48 h did not alter the measured concentrations. These results imply that Fol1 and Fol113 NPs can be degraded and used by gut microbes.

Inulin is a popular dietary polysaccharide that can promote SCFAs-producing gut microbes. We further performed in vitro fermentation in a micro-anaerobic atmosphere, using inulin as positive control to evaluate the direct effect of fullerenol NPs on SCFA-producing bacteria. Inulin significantly enhanced the relative abundance of SCFA-producing bacteria such as *Bifidobacterium* and *Clostridium cluster IV* (Fig. 7c and d), as previously reported [34]. Fermentation with Fol1 and Fol113 NPs also significantly increased the relative abundance of *Clostridium IV* and, to a lesser extent, *Bifidobacterium* (Fig. 7c and d). Another putative SCFA producer, *Allobaculum* spp*.*, was significantly increased by Fol1 and Fol113 in vitro and in vivo, whereas inulin had minimal effect (Fig. 7e). These results suggest that fullerenol NPs possess a similar but not identical capacity to inulin to modulate gut microbiota in vitro.

We also investigated the absorption and excretion of orally administrated fullerenol NPs in mice using positron emission tomography (PET). The microPET images taken 0.5, 1, 2, and 6 h after ^64^Cu-fullerenol administration showed that the signals appeared exclusively in the GI tract (Fig. 7f). The radioactivity level 24 h after oral administration as not detectable by the PET imaging detector, suggesting that Fol1 and Fol113 NPs distributed mainly in the GI tract and were excreted within 24 h of oral administration. We further quantified ^64^Cu-fullerenol excretion in feces and urine at 1, 2, 4, 6, and 24 h after single oral administration (Fig. 7g). The percentages of ^64^Cu-fullerenols in were calculated by using the equation (Σactivity of excrement/total activity). The total excretion was the sum of percentages of ^64^Cu in all the waste (Σ_0-t_urine + Σ_0-t_ feces). Figure 7g shows that ~95% of ^64^Cu-fullerenol was excreted within 24 h. In other words, few NPs were absorbed by the animals. This is further evidence that fullerenol might indirectly regulate lipid metabolism in the GI tract rather than direct effects in the liver or circulation.

Collectively, this set of in vitro experiments suggests that the effects of fullerenols on lipid metabolism could be attributable to their regulation of gut microbiota. Although the underlying mechanism remains to be elucidated, and the diverse structural effects of Fol1 and Fol113 need further investigation, our results indicate that fullerenol NPs might promote SCFA-producing microbes by functioning as their nutrients. Indeed, clinical studies have shown that SCFAs can substantial decrease TG levels but only achieve a modest decrease in cholesterol [35]. In our study, fullerenol NPs differentially affected TC and TG levels, with greater impacts on TG. Therefore, the increased abundance of SCFAs-producing genera and high fecal SCFA content in fullerenol NP-treated animals verify the microbe-regulating effects of fullerenol NPs in vivo.

### Subtly varied surface structures may contribute to the differential effects of Fol1 and Fol113 NPs on the microbiota

Although Fol1 and Fol113 had similar influences on the overall gut microbiota structure and host metabolism, their impacts on special microbes were discrepant. For instance, diversity analysis by Shannon rarefaction OTU estimates, observed species, Chao1 and Shannon diversity indexes indicated that Fol113 NPs significantly decreased the diversity of the gut microbiota while Fol1 showed no influence (Additional file 1: Figure S6a-S6d). On individual taxon, Fol1 selectively enriched the phyla of Verrucomicrobia, while Fol113 decreased its abundance (Additional file 2: Table S3). The relative percentage of *Akkermansia*, the major genus in *Verrucomicrobia* that is known to be beneficial for the prevention and treatment of obesity, dyslipidemia, and diabetes, was increased more than 40-fold in Fol1-treated mice, while there was only a slight increase in Fol113-treated mice (Fig. 2d and Additional file 2: Table S3). While both fullerenol NPs promoted the flourishing of SCFA-producing microbes and increased SCFA production, Fol1 was more effective Fol113 (Figs. 2, 3 and 4). Accordingly, the effect of Fol1 NPs in decreasing TG blood and liver concentrations was greater than that of Fol113 NPs (Fig. 6). We propose that the differential effects of the two fullerenol NPs on special gut microbes and host metabolism might be attributable to subtle variations in their surface structures.

Multiple investigations have demonstrated that NP surface structure profoundly influences their bioactivities [2, 21, 36]. Fullerenols are complicated mixtures of compounds, and their peroxo and hydroxy groups deeply affect their biological activities [3, 37]. Mizuno et al. reported that the in vitro inhibitive effect of fullerenol NPs on microbe growth was markedly enhanced with additional surface hydroxyl groups [24, 31, 38]. The Fol1 NPs in the present paper (C_60_(OH)_7_(O)_8_) had more peroxo or epoxy groups, while Fol113 NPs (C_60_(OH)_11_(O)_6_) had more hydroxy groups. Physiochemical characterization showed that Fol113 NPs were more soluble, more hydrophilic, and formed smaller aggregates in water than Fol1. Therefore, the subtly varied surface structures may contribute to the differential effects of Fol1 and Fol113 NPs on the microbiota.

## Conclusions

Our results provide clear evidence that Fol1 and Fol113 fullerenol NPs can change gut microbiota structure both in vitro and in vivo. They selectively enrich SCFA-producing bacteria and promote SCFA production. The modulating effects of Fol1 and Fol113 NPs on gut microbiota and their metabolites ultimately lead to significant physiological benefits that prevent hyperlipidemia.

## Additional files

Additional file 1: Table S1. The Zeta potential and hydrodynamic sizes of Fol1 and Fol113. **Table S2**. Oligonucleotide primers used in this work. **Figure S1**. Physicochemical characterization of fullerenols. (a) Pictures of respective solutions, (b) UV-vis spectra, (c) FTIR spectra, (d) and (e) XPS spectra of Fol 1 and Fol 113. (f) and (g) MALDI-TOF spectra of Fol 1 and Fol 113. **Figure S2**. The Rarefaction curves (a) and Shannon-Wiener curves (b) indicated a sufficient coverage of the phylotypes by the current sampling number and sequencing depth. **Figure S3**. OTU Venn analysis. **Figure S4**. Responses of top 50 abundant OTUs to fullerenols treatment. (a) Heatmap showing the abundance of top 50 OTUs. (b) Represented abacterial taxa information (phylum, family, genus and species) of 50 OTUs from **a**. White and black circles indicate decreased and increased OTUs, respectively, in the Fol1- and Fol113-treated groups compared to control. Blank indicates similar abundance between fullerenol-treated and control groups. **Figure S5**. Quantitative analysis of fullerenols in gut flora fermentation solution by MALDI-TOF-MS. (a) MALDI-TOF-MASS spectra of samples; (b) the standard curve of C_60_/ C_70_ and the concentration of C_60_. **Figure S6**. Diversity and richness of the gut microbiota in mice. (a) OTU estimates, (b) Observed species, (c) Chao1 diversity index, (d) Shannon diversity index. Data are represented as means ± standard error. Differences were assessed by ANOVA and denoted as follows: ^***^P < 0.001. (DOCX 3554 kb)

Additional file 2: Table S3. Taxon-based summaries for oral administration of fullerenol NPs. (XLSX 152 kb)
