# Supplementary material for: The antihyperlipidemic effects of fullerenol nanoparticles via adjusting the gut microbiota in vivo
Source: Part Fibre Toxicol. 2018 Jan 17;15:5. doi: 10.1186/s12989-018-0241-9 (PMC5773151; doi:10.1186/s12989-018-0241-9)
Supplement: Supplementary file 1 — The Zeta potential and hydrodynamic sizes of Fol1 and Fol113. Table S2. Oligonucleotide primers used in this work. Figure S1. Physicochemical characterization of fullerenols. (a) Pictures of respective solutions, (b) UV-vis spectra, (c) FTIR spectra, (d) and (e) XPS spectra of Fol 1 and Fol 113. (f) and (g) MALDI-TOF spectra of Fol 1 and Fol 113. Figure S2. The Rarefaction curves (a) and Shannon-Wiener curves (b) indicated a sufficient coverage of the phylotypes by the current sampling number and sequencing depth. Figure S3. OTU Venn analysis. Figure S4. Responses of top 50 abundant OTUs to fullerenols treatment. (a) Heatmap showing the abundance of top 50 OTUs. (b) Represented abacterial taxa information (phylum, family, genus and species) of 50 OTUs from a. White and black circles indicate decreased and increased OTUs, respectively, in the Fol1- and Fol113-treated groups compared to control. Blank indicates similar abundance between fullerenol-treated and control groups. Figure S5. Quantitative analysis of fullerenols in gut flora fermentation solution by MALDI-TOF-MS. (a) MALDI-TOF-MASS spectra of samples; (b) the standard curve of C60/ C70 and the concentration of C60. Figure S6. Diversity and richness of the gut microbiota in mice. (a) OTU estimates, (b) Observed species, (c) Chao1 diversity index, (d) Shannon diversity index. Data are represented as means ± standard error. Differences were assessed by ANOVA and denoted as follows: ***P < 0.001. (DOCX 3554 kb) [file 12989_2018_241_MOESM1_ESM.docx]

Supporting Information

**Gut Microbiota Contributes to the Antihyperlipidemic Effects of Fullerenol Nanoparticles with Different Subtly Structures**

Juan Li^1^, Runhong Lei^1^, Xin Li^2^, Fengxia Xiong^1^, Quanyang Zhang^2^, Yue Zhou^2^, Shengmei Yang^1^, Yanan Chang^1^, Kui Chen^1^, Weihong Gu^1^, Chongming Wu^*, 2^, Gengmei Xing^^[[1]](#footnote-1)^*,1^

*1. CAS Key Laboratory for Biomedical Effects of Nanomaterial & Nanosafety, Institute of High Energy Physics, Chinese Academy of Science (CAS), Beijing*

*100049, China*

*2 . Pharmacology and Toxicology Research Center, Institute of Medicinal Plant Development, Chinese Academy of Medical Sciences & Peking Union Medical College, Beijing*

*100193, Ch*ina

Table S1. The Zeta potential and hydrodynamic sizes of Fol1 and Fol113. (n=5)

| Sample | solution | Zeta potential (mV) | Diameter(nm) | PDI |
| --- | --- | --- | --- | --- |
| Fol 1 | Saline  simulated Gastric solution  simulated intestinal solusion | -24.23±1.79  11.14±0.30  -14.23±0.38 | 126.70±15.20  211.05±6.48  31.3±0.72 | 0.38±0.04  0.34±0.02  0.476±0.05 |
| Fol 113 | saline  Gastric solution  simulated intestinal | -18.00±0.77  4.82±0.532  -18±0.43 | 90.70±12.10  178.03±11.55  30.05±0.51 | 0.43±0.09  0.43±0.08  0.31±0.02 |

Table S2. Oligonucleotide primers used in this work.

| Targets | Primers | Sequence (5’-3’) |
| --- | --- | --- |
| All bacteria | Uni331 modF | TCCTACGGGAGGCAGCAGTG |
|  | E533 modR | TTACCGCGGCTGCTGGCACG |
| Clostridium IV | Sg-Clept-F | GCACAAGCAGTGGAGT |
|  | Sg-Clept-R3 | CTTCCTCCGTTTTGTCAA |
| Costridium XIVa | Erec-482-F | CGGTACCTGACTAAGAAGC |
|  | Erec-688-R | GTTCCTCCTAATATCTACGC |
| Bifidobacterium | Bidf-F | CTCCTGGAAACGGGTGG |
|  | Bidf-R | GGTGTTCTTCCCGATATCTACA |
| Allobaculum | Allo-F | ACCTGCGGTGCATTAGYTGG |
|  | Allo-R | GCATYGCTCGTTCAGGCTTGC |
| BcoA transferase | BcoA-F | GYTNGGHATYGGYGGHATGC |
|  | BcoA-R | CCTGCCTTTGCAATRTCNACRAANGC |
| Butyrate kinase | Buk-F | TGYWDKHGTWGGHMGWGGYG |
|  | Buk-R | TCATCTRYAACWACHGGRTCY |


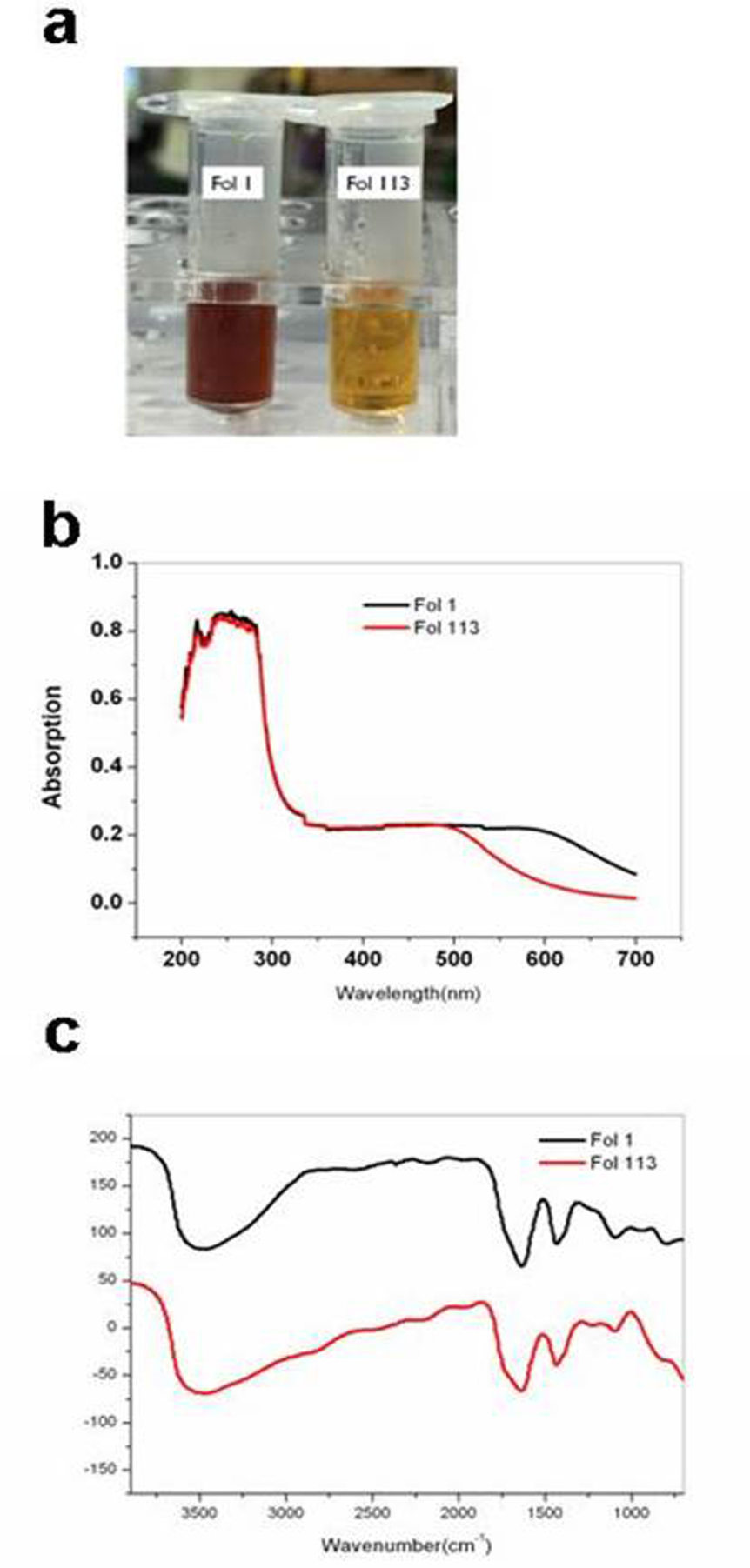


Figure S1. Physicochemical characterization of fullerenols. (a) pictures of respective solutions, (b) UV-vis spectra, (c) FTIR spectra.


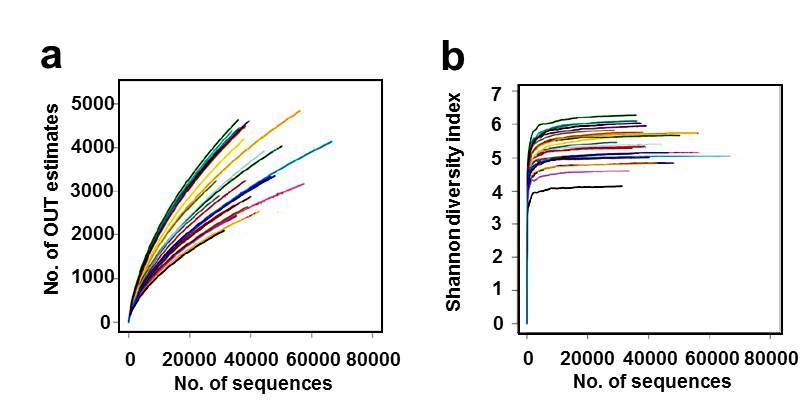


Figure S2. The Rarefaction curves (a) and Shannon-Wiener curves (b) indicated a sufficient coverage of the phylotypes by the current sampling number and sequencing depth.


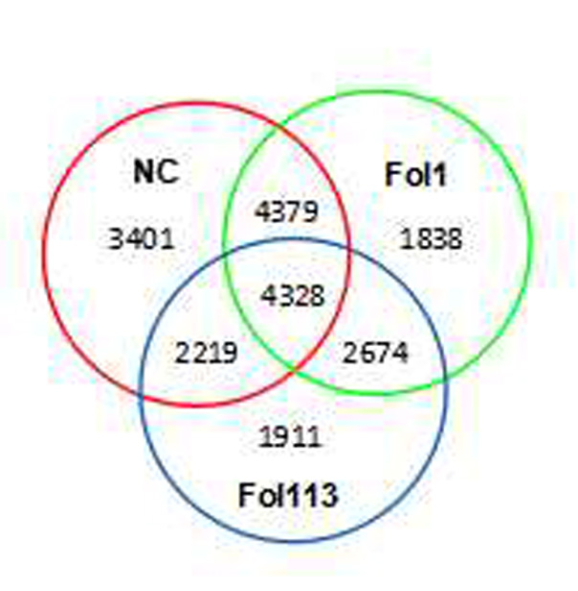


Figure S3. OTU Venn analysis.


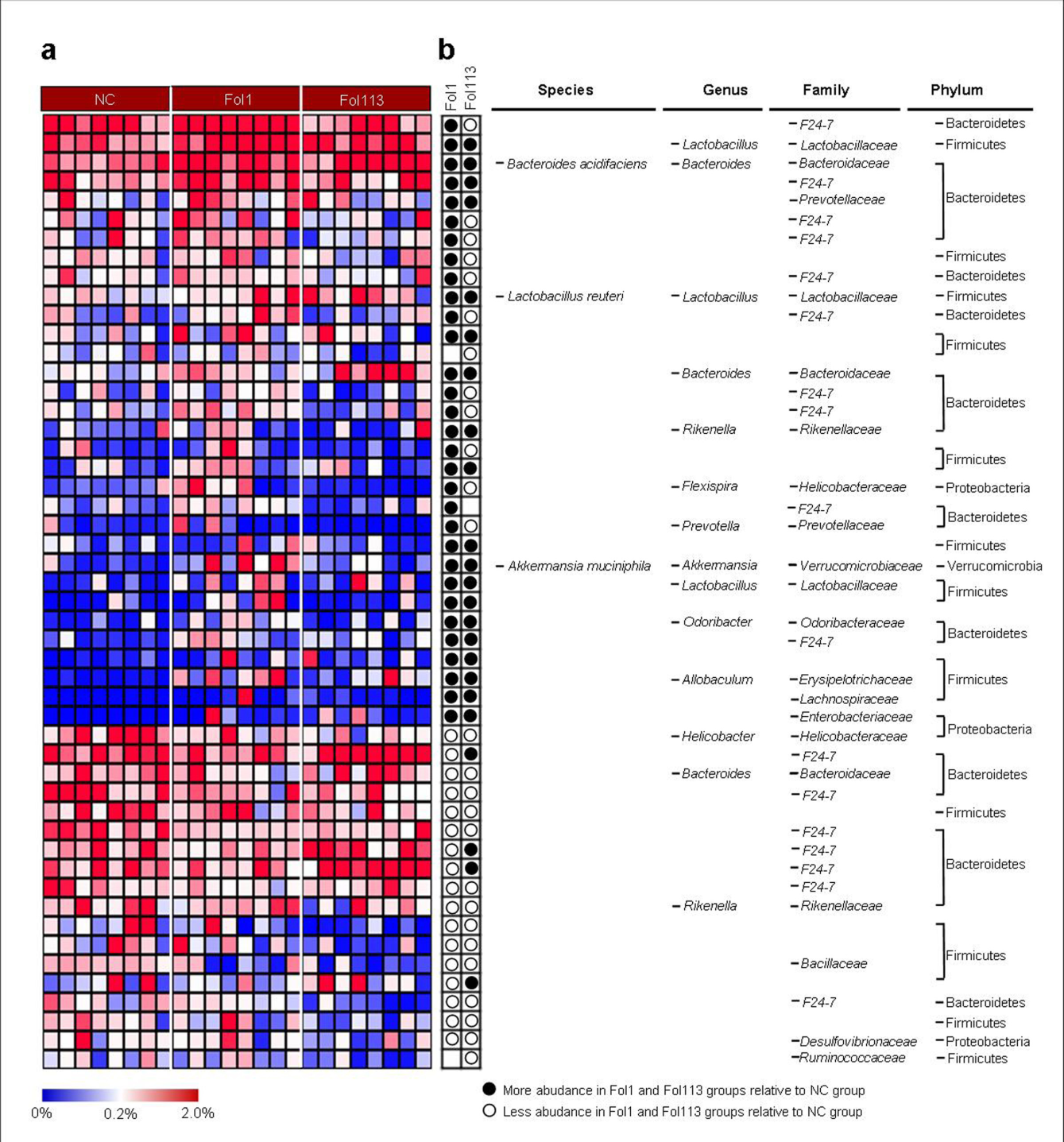


Figure S4. Responses of top 50 abundant OTUs to fullerenols treatment. (a) Heatmap showing the abundance of top 50 OTUs. (b) Represented abacterial taxa information (phylum, family, genus and species) of 50 OTUs from **a**. White and black circles indicate decreased and increased OTUs, respectively, in the Fol1- and Fol113-treated groups compared to control. Blank indicates similar abundance between fullerenol-treated and control groups.


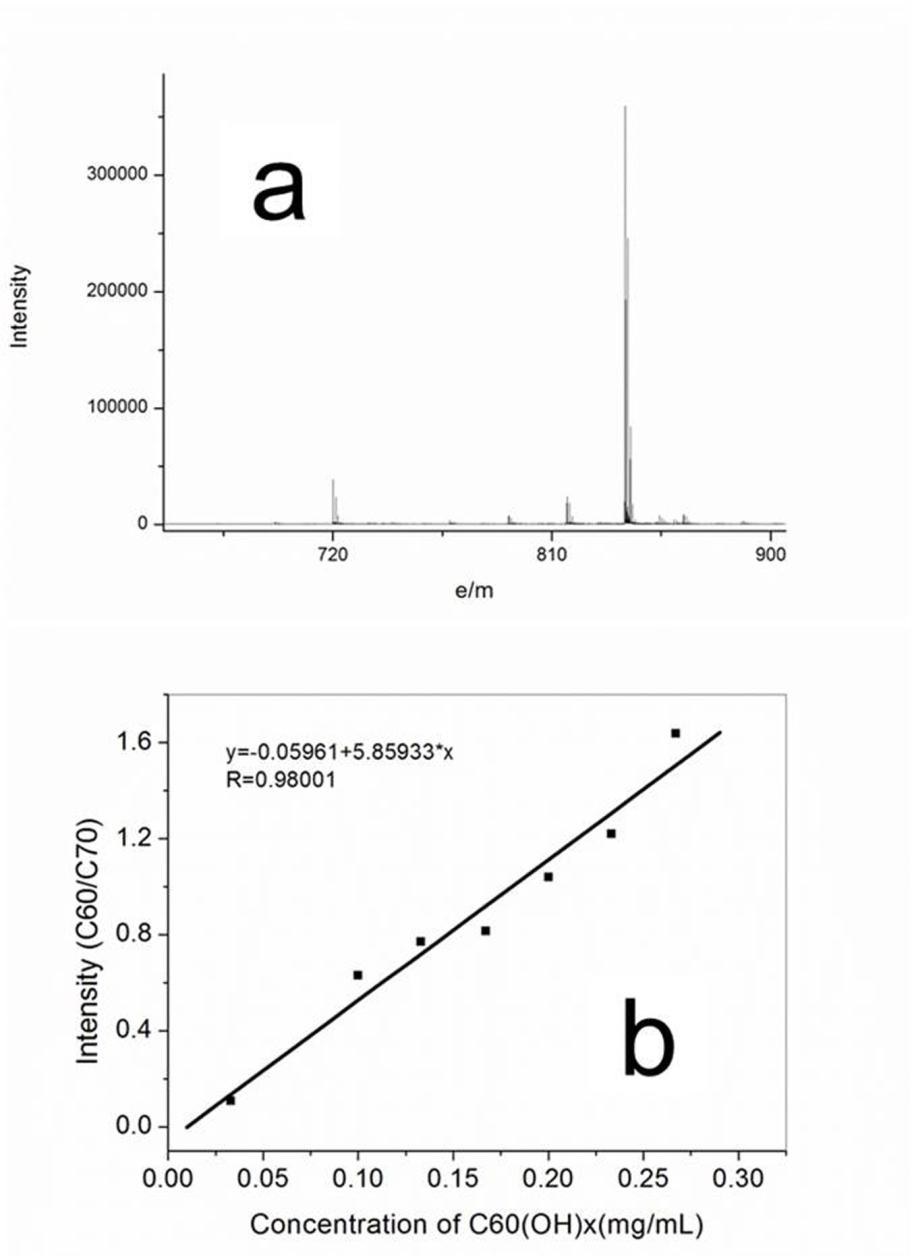


Figure S5. Quantitative analysis of fullerenols in gut flora fermentation solution by MALDI-TOF-MS. (a) MALDI TOF mass spectra of samples; (b) the standard curve of C_60_/ C_70_ and the concentration of C_60_.


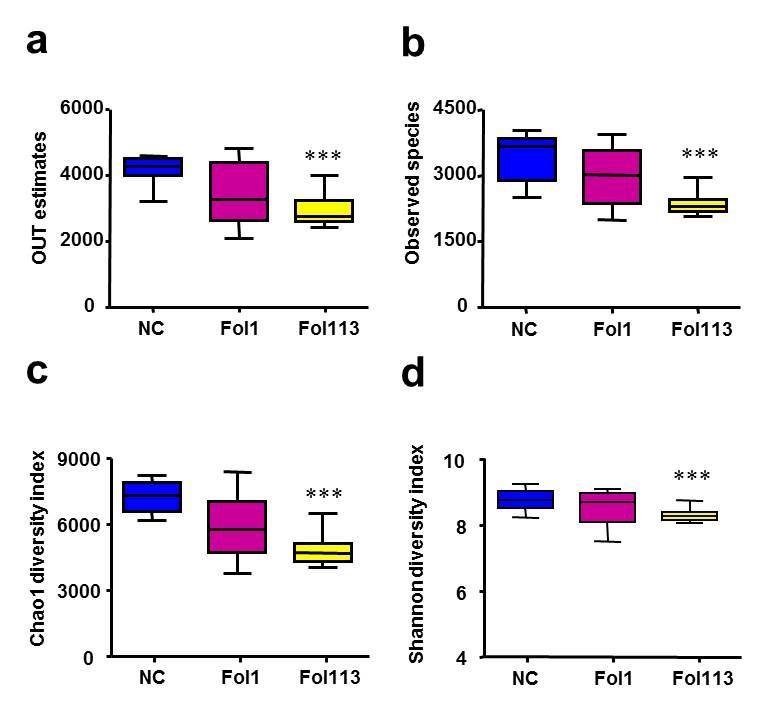


Figure S6. Diversity and richness of the gut microbiota in mice. (a) OTU estimates, (b) Observed species, (c) Chao1 diversity index, (d) Shannon diversity index. Data are represented as means ± standard error. Differences were assessed by ANOVA and denoted as follows: ^***^*P* < 0.001.

1. ^*^ Corresponding authors: E-mails: cmwu@implad.ac.cn (W.C.) and xinggm@ihep.ac.cn (X.G.); Tel/Fax: +86 10 88236456. [↑](#footnote-ref-1)
